# Low Pre-Treatment Count of Circulating Endothelial Progenitors as a Prognostic Biomarker of the High Risk of Breast Cancer Recurrence

**DOI:** 10.3390/jcm8111984

**Published:** 2019-11-15

**Authors:** Piotr Rhone, Kornel Bielawski, Katarzyna Ziołkowska, Danuta Rość, Barbara Ruszkowska-Ciastek

**Affiliations:** 1 Clinical Ward of Breast Cancer and Reconstructive Surgery, Oncology Centre Professor Franciszka Łukaszczyk Memorial Hospital, 85-796 Bydgoszcz, Poland; prhone@wp.pl; 2 Department of Pathophysiology, Faculty of Pharmacy, Nicolaus Copernicus University, Collegium Medicum in Bydgoszcz, Street: Skłodowskiej-Curie 9, 85-094 Bydgoszcz, Poland; kornel-bielawski@wp.pl (K.B.); stankowska_katarzyna@wp.pl (K.Z.); drosc@cm.umk.pl (D.R.)

**Keywords:** circulating endothelial progenitor cells, invasive breast cancer, metastasis, adjuvant treatment, angiogenesis

## Abstract

Neoangiogenesis is mediated by circulating bone marrow-derived endothelial progenitors (circulating EPCs). The aim of the study was the quantification of circulating EPCs from the peripheral blood mononuclear cells of invasive breast cancer (IBrC) patients by flow cytometry, before and after cancer adjuvant treatment. A total of 88 stage IA-IIB primary IBrC patients were enrolled prospectively. Circulating EPCs with the immune-phenotype CD45−CD34+CD133+CD31+ were assessed. Treatment significantly reduced the number of EPCs/µL in the general IBrC cohort. However, there was a relevant elevation in the number of circulating EPCs after nine months of adjuvant treatment in the group of patients aged ≥ 55 years, of T2 clinical type, with nodal involvement (N1) and Ki67 expression > 15%. Follow-up revealed a significantly higher incidence of disease relapse in breast cancer patients with low pre-treatment circulating EPCs levels compared with those with a high baseline circulating EPCs count. The receiver-operating characteristic curve identified a tumour diameter of 2.1 cm as the best cut-off value to discriminate between disease-relapse subjects and non-relapse disease cases. Our study strongly indicates that, next to tumour diameter and Ki67 expression, circulating bone marrow-derived EPCs may serve as useful markers for predicting therapeutic outcomes as well as a future prognosis.

## 1. Introduction

The formation of neovasculature is a crucial step for tumour progression, invasion, and dissemination to distant organs. Neoplasm angiogenesis is a chaotic, uncontrolled proliferation of endothelial cells (EC), but also the de novo creation of irregular and abnormal blood vessels. Tumour spread is the principal cause of death in invasive breast cancer patients. Interestingly, major improvements in the diagnosis and treatment of primary breast cancer did not ameliorate overall patient survival [1], perhaps due to the fact that breast cancer is a complex disease in respect to clinical, histo-pathological, molecular, and genetic points of view; hence, the approach to the patient must invariably be personalised and tailored to the patient’s needs. In order to predict metastases, tumour size, histological grade, nodal status, or specific molecular subtypes have generally been used [2]. However, recently, it has been suggested that circulating endothelial progenitor cells (circulating EPCs) may be a non-invasive biomarker of neoangiogenesis in breast cancer and other diseases [3] and may serve as a strong indicator for predicting a relapse or disease progression [2].

In 1997, Asahara discovered new cells with the ability to incorporate in the foci of physiological or pathological neovascularisation [4]. Bone marrow-derived endothelial progenitors constitute a small, heterogeneous subpopulation of stem cells. EPCs play a fundamental role in endogenous vascular structure renovation and amelioration of blood flow as well as by re-endothelialisation in post-natal angiogenesis [5,6]. In normal physiological conditions, EPCs are quiescent, but in response to a vascular injury or hypoxia, they acquire the ability to circulate in the peripheral blood, proliferate, and differentiate into mature cells with the endothelial phenotype [7,8], e.g., in myocardial ischaemia and infarction, limb ischaemia and wound healing [7], endometrial remodelling following hormone-induced ovulation [4], but also in tumour growth [4,9].

Circulating EPCs can be roughly divided into two subpopulations: Early and late. The main surface antigens of early EPCs include CD34+, CD133+, CD31+, c-kit+, CXCR4+, VEGFR2+, VE-cadherin+, CD146+, and vWF+, while late EPCs markers are: CD34+, CD133−, c-kit−, VEGFR-2+, VE-cadherin+, CD146+, vWF+, CD31+, and CXCR4+. On the surface of both subpopulations, there is a lack of haematopoietic stem cell antigens: CD14 and CD45. Interestingly, marker CD133 is expressed only on the surface of immature EPCs and is lost during maturation into endothelial cells. CD133 expression can also be used to differentiate platelet and endothelial microparticles from circulating EPCs, while CD31 is a typical endothelial antigen [4,5,7]. The CD45− population is ordinarily determined as authentic EPCs with endothelial properties and CD45+ EPCs are part of the haematopoietic lineage [10]. Early EPCs mainly promote a pro-angiogenic pattern via secreting growth cytokines such as hepatocyte growth factor (HGF), vascular endothelial growth factor (VEGF), insulin growth factor (IGF-1), IL-10, and Il-8, but late ones take part in the formation of the endothelium and indirectly enhance tubulogenesis [5].

The number of CD34+ cells that co-express CD309, CD31, and CD133 in peripheral blood in the healthy population is around 0.4% ± 0.2% of the total CD34+ population (0.002% of total mononuclear cells). Quantification of circulating EPCs is quite complicated due to the low count of cells in the peripheral blood, methodological divergencies, and a lack of agreement on phenotypic identification, since circulating EPCs cannot be effectively determined by a single surface antigen [7]. However, the pathophysiological role of circulating EPCs in many cancers is of interest to many research groups. In our previous study, we reported that circulating EPCs were significantly higher in breast cancer patients and the level was inversely associated with the expression of the proliferation marker (Ki67) and the histological grading of the breast cancer [9,11]. It has been established that circulating EPCs counts might either elevate or even drop after surgery [12,13]. These divergent results can mainly be explained by the different types of surgical procedures which were used. 

Little is known about the fluctuations of circulating endothelial progenitors counts during adjuvant treatment of IBrC. The aim of this study was to document the different effects of adjuvant therapy on circulating EPCs, which are considered important biomarkers indicative of neovascularisation. According to our previous studies, we suggest that alterations in the number of circulating EPCs in patients with invasive breast cancers can indicate the advancement of the malignancy [9,11] but in the current study, we aimed to explore whether the fluctuation in the circulating EPCs count can predict treatment response and disease recurrence. 

## 2. Methods

### 2.1. Patient Samples and Clinical Data

Eighty-eight consecutive histologically confirmed primary, M0, unilateral, invasive breast carcinoma (IBrC) patients with a median age of 54.4 years (range 41–66 years) were enrolled in this prospective, single-centre study, from November 2015 to January 2018. First, selection of early-stage IBrC patients who were naïve of chemotherapy was an entry criterion for our study. All patients were of Polish descent. All patients were treated surgically with curative intent by conserving (BCS) surgery (71 patients; 81%) or modified radical mastectomy (MRM) (17 cases; 19%) and adjuvant external beam radiation, chemotherapy, immunotherapy, and/or hormonal therapy. There was no perioperative mortality and the average length of hospital stay was 7.1 ± 1.3 days. The subjects were under the care of the health professionals from the Clinical Ward of Breast Cancer and Reconstructive Surgery, Oncology Center Prof. F. Łukaszczyk Memorial Hospital, Bydgoszcz, Poland. 

The study was performed under the appropriate University ethics approvals and in accordance with the principles embodied in the Declaration of Helsinki (permission no. KB 547/2015). All subjects provided written informed consent to participate in this study after a full explanation of the study had been given.

The disease stages were confirmed based on the tumour, node, and metastasis (TNM) classification of the International Union Against Cancer and AJCC 7th Edition Staging for Breast Cancer. Forty-four (50%) patients developed stage IA (T1N0M0—T1 tumours without nodal micrometastases) and 44 subjects (50%) had stage IIA-IIB (T1N1M0—T1 tumours with nodal micrometastases—or T2N0M0/T2N1M0—T2 with/without nodal micrometastases), and the median diameter of the tumour was 1.69 cm (range 0.5–3.5 cm). The histological type was determined according to the classification of the World Health Organization. Eight (10%) patients had an invasive lobular carcinoma and 80 (90%) cases developed an invasive ductal carcinoma. Tumours were graded according to the Elston–Ellis grading system, in order to stratify breast cancer. Well-differentiated cells (low grade) were reported in only four cases (4.5%), intermediate grade (moderately differentiated cells) were identified in 66 patients (75%), and, finally, high grade (poorly differentiated cells) were noted in 18 patients (20.5%). The molecular subtypes of IBrC were characterised based on immunohistochemical (IHC) evaluation of hormonal receptor (HR: oestrogen receptor—ER; progesterone receptor—PR), human epidermal growth factor receptor 2 (HER2), and Ki67− mitotic index expressions. Among the breast cancer patients, 53 (60%) had luminal A subtype (ER+PR+HER2−, Ki67 < 14%), luminal B HER2 negative (ER+PR+/−HER2−, Ki67 > 14%) was noted in 16 cases (18%) with luminal B HER2 positive (ER+PR+/−HER2+, Ki67− all values) diagnosed in seven women (8%). Non-luminal HER2 positive (ER−PR−HER2+, Ki67− all values) was recognised in three patients (4%) and a basal-like subtype (BLBC) (ER−PR−HER2−, Ki67− all values) was reported in nine subjects (10%). Lymph nodes involvements, N1, were noted only in 22 (25%) cases. Invasive breast cancer was diagnosed in the left breast in 45 cases (51%) and right-sided breast tumours were noted in 43 patients (49%). The main assumption of this study was to create a homogeneous group of patients with invasive breast cancer at the early stage without co-morbidities. However, due to the qualification of perimenopausal women, it was impossible to eliminate basic lifestyle-related diseases including obesity and hypertension. There were 20 (23%) hypertensive patients and 14 obese subjects (16%), all of whom had a body mass index (BMI) lower than 40 kg/m^2^. BMI was calculated as weight in kilograms divided by height squared in metres. BMI (kg/m^2^) was categorised as normal (18.5–24.9), overweight (>25.0–29.9), and obese (>30.0) according to WHO recommendations. Height and weight were measured to the nearest 0.1 cm and 0.1 kg, respectively. Hypertension was identified according to the values of a systolic blood pressure (BP) of 140 mmHg or higher and a diastolic BP of 90 mmHg or higher. According to their menopausal status, patients were divided into premenopausal and postmenopausal cases. Natural menopause was defined as permanent cessation of menstruation for at least 12 months. Thirty women (34%) were premenopausal and 58 patients (66%) were postmenopausal. 

### 2.2. Exclusion Criteria

Patients with an in-situ carcinoma, bilateral IBrC, advanced breast cancer with metastasis, as well as with neoadjuvant chemotherapy and radiotherapy were not recruited to this study. Additionally, subjects with overt diabetes mellitus (DM) or impaired glucose tolerance, recent bleeding or thrombotic events, other cancers, recent surgery, or trauma did not qualify for the study. None of the patients received concurrent medication known to influence the mobilisation of circulating endothelial progenitors from the bone marrow (i.e., hydrocortisone, nonsteroidal anti-inflammatory drugs, insulin, or statins) at the time of blood sampling.

### 2.3. Treatment Patterns

All patients were treated according to standard guidelines adopted from the recommendations published by the National Comprehensive Cancer Network (NCCN) Guidelines for Practice. Seventy-one underwent BCS and 17 had radical mastectomy. Adjuvant chemotherapy regimens were instituted in 43 (49%) women, 17 with and 26 without lymph node involvement. Adjuvant chemotherapies were anthracycline-containing (*n* = 33) and non-anthracycline (*n* = 10) containing drugs; indicated from 4 to 6 cycles. Ten HER2-positive patients (11%) were required to receive an adjuvant combining trastuzumab (a humanised anti-HER2 monoclonal antibody) with chemotherapy. The chemotherapy regimens and dosages depended on the doctors’ recommendations. Per protocol, radiotherapy had to start within 1–2 weeks after completion of the adjuvant chemotherapy. Generally, in the study group, a dose of 42.5 gray (Gy) was delivered in 17–20 fractions over 4–6 weeks to the chest wall applying tangential photon fields, and for subjects with N1 status, to the supraclavicular, infraclavicular, and axillary nodes using an anterior field matched to the tangential fields. Forty-five (63%) breast-conserved patients received, in addition, a sequential boost of 10 Gy delivered in five fractions to the initial tumour bed using a direct electron field. Only 14 patients did not require adjuvant radiotherapy (16%). Five women with node-negative disease underwent adjuvant endocrine therapy only (tamoxifen or aromatase inhibitor). Fifteen cases were free of any hormone therapy due to the small size of the tumour or basal-like subtype of IBrC. None of these patients received treatment with erythropoietin or granulocyte monocyte colony stimulating factor (GM-CSF). 

### 2.4. Patient Follow-Up Information 

Follow-up was completed in 88 patients. For the progression-free survival analysis, 11 events occurred and follow-up ranged from 13 to 40 months (the median follow-up was 32.5 months) with a 12.5% recurrence rate. Follow-up times were calculated from the date of the initial clinic visit until the earliest event of interest, i.e., disease spread, death, or the last date of contact as of the end of February 2019 and were expressed in months. For each patient, follow-up visits at the medical centre were performed four and nine months after surgery.

### 2.5. Blood Collection

Blood samples from patients were collected three times. The first blood collection occurred 24 h prior to any treatment (I—baseline values). The second blood specimen collection took place four months after the tumour removal procedure (II) in order to estimate the alteration in the number of circulating EPCs after surgery and during radiotherapy or chemotherapy. However, the third blood sample (III) was collected a maximum of three months after the last cytotoxic infusion and nine months after tumour resection to avoid the direct effects of chemotherapy or surgical wound healing on the circulating EPC numbers. 

### 2.6. Flow Cytometry

Preoperative and postoperative blood samples were taken from a peripheral venipuncture by a fresh needle insertion; arterial and central lines were not used. This consisted of the following: Blood was drawn by venipuncture into BD Vacutainer^®^ Plus Plastic K2EDTA tubes containing potassium ethylene-diaminetetraacetic acid in order to measure the number of circulating EPCs. Blood was obtained according to clinical standards. Quantification of circulating EPCs from peripheral blood by fluorescent-activated cell sorting (FACS) analysis (Becton Dickinson, San Diego, CA, USA) was performed according to a protocol and recommendations provided by the by Mancuso et al. and our former studies [9,14,15]. Whole blood stains were performed within two hours after blood collection to minimise cell death. The acquired data were analysed using CellQuest software (Becton Dickinson). 

**Figure 1:**
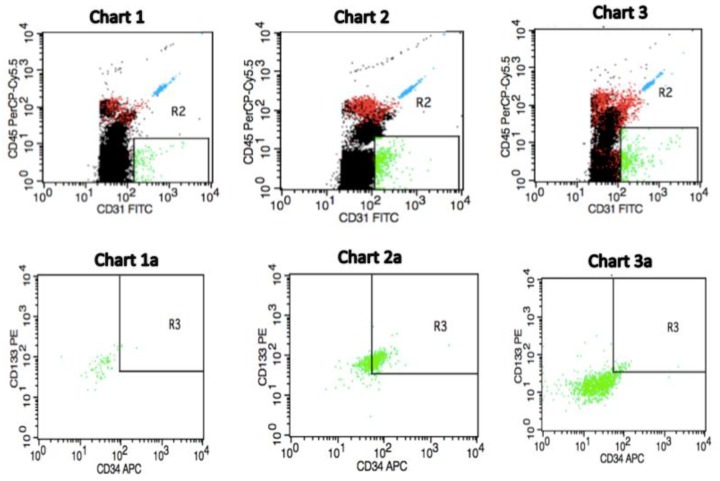
Sample of selected flow cytometric plots for identification of circulating endothelial progenitor cells depending on pre-treatment counts in invasive breast cancer cases and in a healthy individual. Charts 1 and 1a demonstrate a profile of a subject free of breast cancer (control) with circulating endothelial progenitor cells (EPCs) count of 0.30 cells/µL. Charts 2 and 2a show an invasive breast cancer (IBrC) patient with T1a, N0, M0, grade = 2, tumour diameter = 0.5 cm, ER/PR+ and HER2-Ki-67 = 15%, a baseline number of circulating EPCs was 37.99 cells/µL. A patient after 40 months of follow-up is still alive. Charts 3 and 3a demonstrate an IBrC subject with T2, N0, M0, grade = 3, tumour diameter = 2.5 cm, ER+, PR−, HER2−; Ki-67 = 45%, a pre-treatment number of circulating EPCs was 4.32 cells/µL. A patient passed away due to lung, liver, and bones metastases. Quantification of circulating EPCs was made in a peripheral blood mononuclear cell (PBMC) fraction. A gating strategy for the identification of circulating EPCs by applying CD45-PerCP-Cy5.5, CD31-FITC (R2: CD45–/CD31+ gating to exclude lymphocytes), CD34-APC, and CD133-PE (R3: CD133+/CD34+ gating identifies endothelial progenitor cells) conjugated antibodies was used. The CD45 population was gated on the overall lymphocyte + monocyte cells in the PBMC. A detailed flow cytometry plots presentation was published in our previous study [9].

For circulating EPCs enumeration, 50 µL of whole peripheral blood was incubated with a 10 μL fluorescein isothiocyanate (FITC)-conjugated anti-CD31, a 10 µL PerCP-Cy5.5-conjugated anti-CD45, as well as a 10 μL APC-conjugated anti-CD34 antibody (all BD Biosciences, Pharmingen, San Diego, CA, USA) and a 20 μL phycoerythrin (PE)-conjugated anti-CD133 antibody (Miltenyi Biotec, Bergisch Gladbach, Germany). Undiluted samples were stained with antibodies for 20 min in the dark. Then, erythrocytes were lysed using a 500 μL lysing solution (BD Biosciences) as well as being added in order to dilute the sample. The solution was incubated for 10 min (Figure 1). The total cell count was determined by TruCount tubes (BD Biosciences, San Jose, CA, USA) containing a calibrated number of fluorescent beads, and “lyse-no-wash” methods were applied to improve the sensitivity [9]. For a clear analysis, at least 100,000 total events were collected by flow cytometry, per sample, to analyse the number of circulating EPCs. The cell densities were calculated as the total number of circulating EPCs per μL blood. Circulating endothelial progenitor cells were defined as negative for the haematopoietic marker CD45 and positive for the early haematopoietic marker of stem cells CD133, which is highly expressed on immature EPCs but is lost when they differentiate into mature endothelial cells [6], as well as positive for the endothelial cell markers CD31 and CD34, which show expression on early haematopoietic and vascular-associated tissue. The measurements were performed by one diagnostician who was blinded to the clinical data of the patients. This ensures relative homogeneity among the samples used in a given study and minimises the differences in sample analyses. 

The number of circulating endothelial progenitor cells in the peripheral blood of healthy postmenopausal women was established in our previous study at 0.36 cells/μL, but the median number of circulating EPCs for invasive breast cancer patients was 10.57 cells/μL [9] (Figure 1).

### 2.7. Immunohistochemical (IHC) Detection of Oestrogen Receptors (ER), Progesterone Receptors (PR), HER2 and Ki67 Expression, and Scoring Method

All surgical specimens were fixed with 10% formalin and embedded in paraffin wax to be used for the immunohistochemical detection of all molecular determinants. ER, PR, and Ki-67 indexes were scored according to the St. Gallen International Breast Cancer Conference guidelines. ER and PR receptors were scored as negative/positive when no/any staining was present in the tumour. The Ki67 proliferative index in the surgical specimens was assigned by the pathologist on the basis of the percentage positive on at least 500 neoplastic cells counted in the peripheral area of the nodule. A cut-off value of 14% was used in all association analyses: When the Ki-67-labelling index was >15%, it was considered high, whereas when Ki-67 was ≤14%, it was considered low. HER2 was scored as 0, 1+, 2+, or 3+ in accordance with the American Society of Clinical Oncology (ASCO) and the College of American Pathologists (CAP): 0, no staining noted or membrane staining that was deficient or faint/barely detectable in ≤10% of tumour cells; 1+, incomplete membrane staining that is poor/barely perceptible within >10% of tumour cells; 2+, circumferential membrane staining that is incomplete and/or weak/moderate within >10% of tumour cells, or complete and circumferential membrane staining that is intense within ≤10% of tumour cells; and 3+, circumferential membrane staining that is complete and intense within >10% of tumour cells. However, scores of 2+ were considered as equivocal samples, which were further analysed for fluorescence in situ hybridisation (FISH) confirmation, which detects gene amplification. FISH was performed using a dual HER2/Cep17 probe. Detailed methodology has been published in our previous work [9,11].

### 2.8. Statistical Analysis

Statistical analysis was performed with Statistica software, version 12.5 (StatSoft, USA). The normality of the variables was tested by the Shapiro–Wilk test. Data are presented as median and interquartile ranges (IQR). The data were compared by means of a non-parametric post hoc ANOVA-Friedman test for more than two dependent variables. The progression-free survival (PFS) time was calculated from the date of enrolment until relapse or the progression of disease. PFS curves were calculated by the Kaplan–Meier method, and the significance level was assessed according to the log-rank test. Cox proportional hazards analysis was used to evaluate the association between clinical variables and survival times. A receiver-operating characteristics (ROC) curve for circulating EPCs count and Ki67 expression and tumour diameter was designed. The strength of association was determined by ROC analysis and expressed in terms of the area under the ROC curve (AUC^ROC^). The AUC was calculated in order to estimate the diagnostic accuracy. Optimal cut-off values were determined. A probability (*p* < 0.05) was considered to be statistically significant.

## 3. Results

The cohort included 88 patients with completely resected early-stage (IA-IIB) invasive breast cancer. The mean age at diagnosis of our sample population was 54.4 years. The relative frequency of postmenopausal women with IBrC was 66% (58 out of 88), and premenopausal women comprised 34% (30 out of 88) of the study population. Pathological grade 2 was high at 75% (66 out of 88) and significantly different from the distribution of other grades. Invasive ductal carcinoma (IDC) was the predominant histopathological subtype of breast cancer at 90% of the studied population. A total of 33 (38%) patients received an anthracycline-based chemotherapy regimen and 10 (11%) received non-anthracycline containing drugs. 

We determined the number of circulating EPCs as CD45−CD34+CD133+CD31+ cells (green dots) in the peripheral blood by flow cytometry. Figure 1 shows a representative flow cytometric analysis from pre-treatment invasive breast cancer patients as well as from a control individual with respect to the baseline circulating EPCs count. We observed that the pre-treatment number of circulating EPCs can predict future outcomes for IBrC patients. Thus, patients with a high baseline number of EPCs exhibit longer survival rates than patients with a low pre-treatment circulating EPCs count. In Figure 1: Charts 1 and 1*a* present a profile of a postmenopausal subject free of breast cancer (control) and other co-morbidities with BMI = 22.84 kg/m^2^, age = 57 years and circulating EPCs count of 0.30 cells/µL, as a representative case. Charts 2 and 2*a* show a postmenopausal IBrC patient with a high baseline number of circulating EPCs with selected clinical and anthropometric features: BMI = 27.28 kg/m^2^, age = 66 years, the patient gave birth to one child, without co-existing diseases, T1a, N0, M0, invasive ductal carcinoma, histological grade = 2, tumour diameter = 0.5 cm, ER/PR+ and HER2-Ki-67 = 15%, number of circulating EPCs = 37.99 cells/µL. A patient after 40 months of follow-up is still alive. On the other hand, charts *3* and *3a* demonstrate a postmenopausal IBrC subject with a low baseline number of circulating EPCs with a clinical profile: BMI = 28.21 kg/m^2^, age = 52 years, the patient gave birth to one child, without co-existing diseases, T2, N0, M0, invasive ductal carcinoma, histological grade = 3, tumour diameter = 2.5 cm, ER+, PR−, HER2−; Ki-67 = 45%, the number of circulating EPCs was 4.32 cells/µL. However, this patient with a low baseline number of circulating EPCs passed away due to lung, liver, and bones metastases within 18 months of diagnosis.

**Figure 2:**
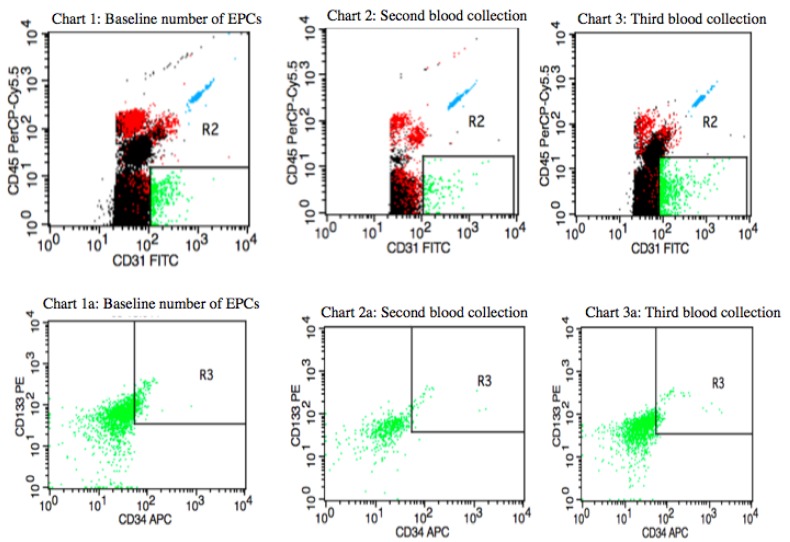
Quantification of circulating EPCs in blood mononuclear cells from invasive breast cancer patient with high pre-treatment level of circulating EPCs with a clinical profile: T1c, N1, M0, grade = 3, tumour diameter was 1.8 cm, ER/PR-HER2-Ki-67 = 10%. A baseline number of circulating EPCs was 22.6 cells/µL (first blood collection); during a second blood collection, the number of circulating EPCs dropped to 3.52 cells/µL; during a third blood collection, the number of circulating EPCs increased to a level of 10.05 cells/µL. The patient, after 40 months of follow-up, is still alive.

Figure 2 shows the distribution of circulating EPCs in a postmenopausal IBrC patient with a baseline number of circulating EPCs equal to 22.6 cells/µL (first blood collection). After four months of the adjuvant treatment, the number of circulating EPCs dropped to a level of 3.52 cells/µL (second blood collection) and after nine months of the treatment the number of circulating EPCs elevated to a level of 10.05 cells/µL (third blood collection). The patient was characterised with selected clinical, anthropometrical features: BMI = 27.89 kg/m^2^, age = 61 years, she gave birth to two children, suffered from hypertension, T1c, N1, M0, stage IIA, invasive ductal carcinoma, high histological grade (G3) according to the Elston–Ellis classification, tumour diameter was 1.8 cm, molecular profile: ER/PR-HER2−; and proliferative index- Ki-67 = 10%, molecular subtype: basal-like subtype (BLBC). The subject was qualified for breast-conserving surgery (BCS). The patient received four cycles of Adriamycin (Doxorubicin) and Cyklofosfamid and then 12 cycles of Paclitaxel and 17 fractions of radiotherapy of a dose of 42.5 Gy. She started chemotherapy one month after diagnosis, which then took five months and, subsequently, one week later she began six weeks of radiotherapy. All adjuvant treatment lasted for eight months; hence, the third blood collection took place one month after completion of the treatment. The patient, after 40 months of follow-up, is still alive. Even though the basal-like subtype IBrC present negative prognosis in this case, the treatment was successfully completed.

**Figure 3:**
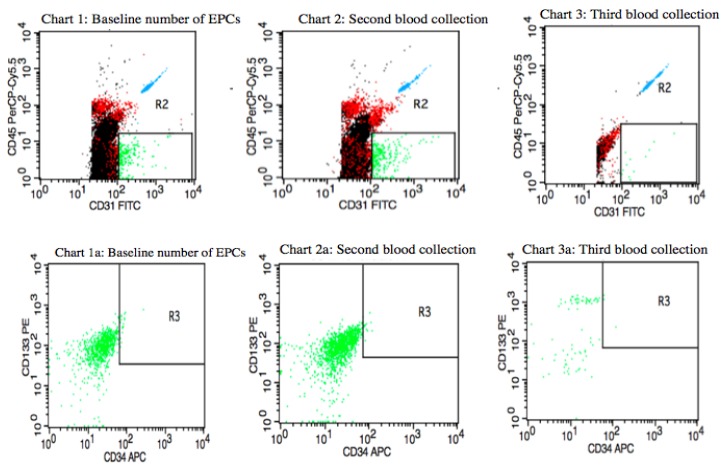
Quantification of circulating EPCs in blood mononuclear cells from invasive breast cancer patients with low pre-treatment level of circulating EPCs with a clinical profile: T1c, N0, M0, grade = 2, tumour diameter was 1.6 cm, ER/PR+HER2−Ki-67 = 5%. A baseline number of circulating EPCs was 2.21 cells/µL (first blood collection); during a second blood collection, the number of circulating EPCs dropped to 1.61 cells/µL; during a third blood collection, the number of circulating EPCs decreased to a level of 0.29 cells/µL. The patient, after 27 months of follow-up, developed a metastasis opposite axillary lymph nodes.

Figure 3 presents variations in circulating EPCs counts in a premenopausal IBrC patient with a baseline number of circulating EPCs equal to 2.21 cells/µL; after four months, the number of circulating EPCs was 1.61 cells/µL. Between the first and second blood collections, the patient underwent adjuvant radiotherapy with 17 fractions of a dose of 42.5 Gy/g, which took four weeks. In the third blood collection, the number of circulating EPCs dropped to a level of 0.29 cells/µL. The patient was characterised with selected clinical and anthropometric features: BMI = 23.98 kg/m^2^, age = 41 years, she gave birth to one child, without co-existing diseases, T1c, N0, M0, stage IA, invasive ductal carcinoma, histological grade (G2) according to the Elston–Ellis classification, tumour diameter = 1.6 cm, ER/PR+HER2− and low proliferative index; Ki-67 = 5%, molecular subtype: luminal A. The subject was qualified for breast-conserving surgery (BCS). She did not receive chemotherapy, only hormone therapy: Tamoxifen as a systemic treatment. The patient, after 27 months of follow-up, developed a metastasis opposite axillary lymph nodes.

In the present study, we examined the potential advantage of circulating EPCs as a marker for breast tumour progression, angiogenesis, and prognosis. Baseline circulating EPCs counts were examined according to the response to adjuvant treatment. Treatment significantly reduced the number of EPCs/μL of peripheral blood in the general IBrC cohort, *p* = 0.0004. Interestingly, according to the second blood collection, in all analyses, the circulating EPCs count was lower with respect to pre-treatment levels. It is worth noting that the number of circulating EPCs did not reach a significant level in premenopausal women with normal weight and of T2 clinical type. However, there was a relevant elevation in the number of circulating EPCs after nine months of treatment in the group of patients aged ≥ 55 years, of T2 clinical type, with nodal involvement (N1), and Ki67 expression > 15%. It should be emphasised that that the number of circulating EPCs were even higher in the third blood collection as compared to baseline values in patients with a pre-surgery expression of Ki67 above 15% (*p* = 0.0183) (Table 1). 

**Figure 4:**
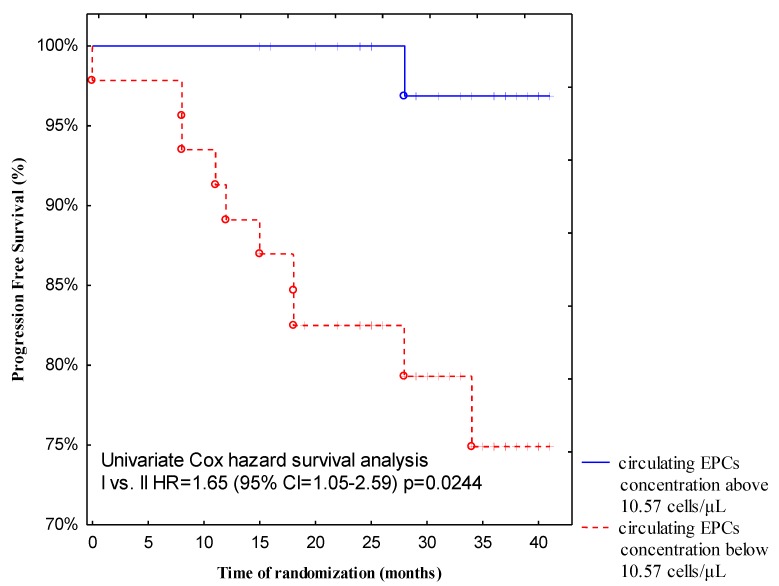
Kaplan–Meier curve for the progression-free survival (PSF) analysis of the entire population with IBrC according to pre-treatment circulating EPC numbers, which were determined with the immune-phenotype CD45−CD34+CD133+CD31+ by flow cytometry. Cut-off values between low 0–10.57 cells/µL (red line) and high > 10.57 cells/µL (blue line) pre-treatment circulating EPC numbers were established. The cut-off value of circulating EPCs count was established at 10.57 cells/μL, due to our previous findings, where we noted that the median value of circulating EPCs was 10.57 cells/μL in breast cancer patients as compared to healthy postmenopausal women as well as according to receiver-operating curve (ROC) analysis [9].

All patients in this study received regular follow-up for 13 to 40 months (median follow-up, 32.5 months) after discharge. For the progression-free survival analysis, 11 events occurred with a PFS rate of 12.5%. Two (2.28%) patients died during the follow-up period due to systemic metastatic disease. Both subjects presented with a low baseline circulating EPCs count (5.83 cells/L and 4.32 cells/L, respectively). More importantly, follow-up revealed a significantly higher incidence of disease relapse from breast cancer patients with low pre-treatment circulating EPCs levels compared with those with high baseline circulating EPCs levels (*p* = 0.0244). Forty-seven patients (53%) demonstrated a low baseline number of circulating EPCs, whereas 41 patients (47%) had a higher pre-treatment circulating EPCs count. Recurrence of the disease in the group of patients with a low pre-treatment number of circulating EPCs occurred in 10 out of 47 (21.27%), but in the group with a high baseline number of circulating EPCs, only 1 out of 41 (2.44%) cases had a recurrence of the disease. We postulate that a low baseline number of circulating EPCs could be predictive of shorter progressive-free survival (Figure 4). 

**Figure 5:**
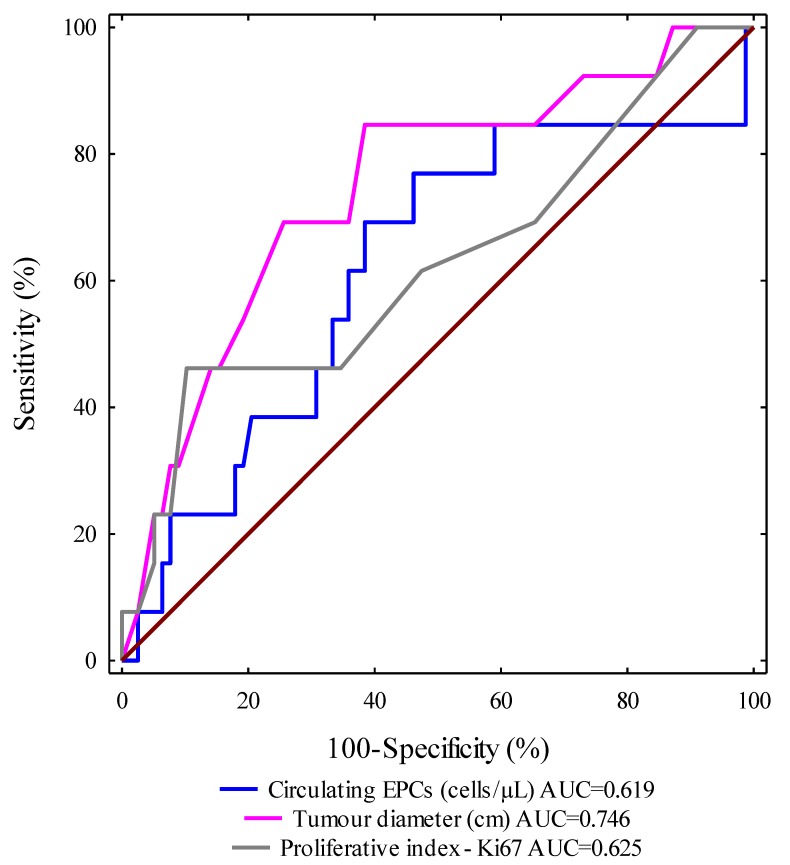
A graph showing three ROC curves for the number of circulating endothelial progenitor cells (EPCs), tumour diameter, and Ki67 expression with different values of area under the curve. The most accurate indicator for disease recurrence presents an area of 0.746 under the curve, which was reached for tumour diameter (*p* = 0.0001).

ROC curves for selected clinical and molecular parameters were designed and the areas under the curves with a 95% confidence interval were calculated (AUC, 95% CI thresholds with sensitivity (SE) and specificity (SP)). We evaluated the ROC curves to assess the diagnostic accuracies of the investigated variables for the prediction of progression-free survival in IBrC subjects. Importantly, the highest level of discrimination was found for the tumour diameter (area under the curve (AUC^ROC^) = 0.746, *p* = 0.0001). The receiver-operating characteristic curve identified a tumour diameter (cm) of 2.1 cm, with 74.4% specificity and 69.2% sensitivity, as the best cut-off value to discriminate between relapse disease subjects and non-relapse disease cases. We observed that the tumour diameter (cm) area under the ROC curve was higher than the areas for circulating EPCs (0.619) and Ki67 expression (0.625). It is worth noting that the AUCs^ROC^ for all analysed parameters were significantly larger in comparison to AUC 0.5 (the borderline for the diagnostic usefulness of the test). Based on the receiver-operating characteristic curve, cut-off points were provided for other determinants in order to distinguish between the relapse disease group and the non-relapse disease cases. Thus, for circulating EPCs, count the value was 10.55 cells/μL with 51.7% specificity and 64.4% sensitivity; the cut-off value for Ki67 expression was 15% with 61.5% specificity and 52.6% sensitivity (Figure 5).

**Figure 6:**
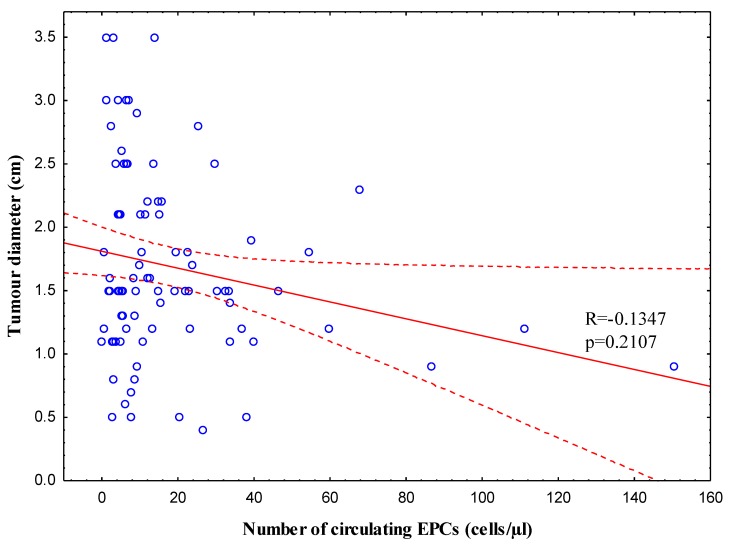
Scatter plot shows a lack of correlation between a pre-treatment number circulating endothelial progenitor cells (EPCs) with tumour diameter (Spearman correlation coefficient, *R* = −0.1347; *p* = 0.2107).

Finally, Figure 6 demonstrates a lack of correlation between the number of circulating EPCs and tumour diameter.

The results obtained by the regression model are summarised in Table 2, demonstrating that pre-treatment circulating EPCs count, tumour diameter, and adjuvant endocrine therapy were independent predictors of PFS (overall model fit: *p* = 0.0092). The results were confirmed by multivariate Cox proportional hazards analyses using the same cut-off points for circulating EPCs. Thus, patients with pre-treatment circulating endothelial progenitors within the range 0–10.57 cells/μL had shorter progression-free survival (recurrence rate: 22%) than patients with a pre-treatment circulating EPCs number above 10.57 cells/μL (relapse rate: 2.4%) with OR = 0.07 (0.01–0.67), *p* = 0.0194. A small tumour diameter (recurrence rate: 3.4%) predicts longer PFS than a tumour with a diameter > 2 cm (recurrence rate: 28.6%). A similar dependency was observed during ROC analysis with respect to tumour diameter. Moreover, patients who were administered hormonal therapy (tamoxifen or aromatase inhibitors) had longer PFS (recurrence rate: 10.7%) than subjects without endocrine treatment (recurrence rate: 30.77%)

## 4. Discussion

This study analysed different clinico-pathological factors contributing to fluctuations in the number of circulating EPCs under the influence of adjuvant treatment in treatment-naïve patients with stage IA-IIB of IBrC, and assessment of the impact of the baseline circulating EPCs number on cancer recurrence has been made. The approach of the current study was to use four concurrent markers (CD45–CD34+CD133+CD31+) to increase the accuracy of detecting circulating EPCs. It is well-known that several factors have been found to be significantly associated with the number of circulating EPCs. Among these factors, ageing, male gender, smoking, drugs, growth factors, or cardiovascular disease have been associated with a reduced level of circulating EPCs in the peripheral circulation [16]. Additionally, in late-stage cancer patients, numerous factors associated with cancer status might affect circulating EPC counts. Hence, elimination of patients with late-stage IBrC allowed us to investigate specifically the association between stage IA-IIB of IBrC and circulating EPCs, regardless of crucial confounders. 

The current armamentarium for cancer treatment includes surgery, which is usually used as the primary course of cancer treatment, and then adjuvant patterns such as radiotherapy along with chemotherapy, hormone therapy, and immunotherapy. It has been proven that thanks to this approach, it is possible to improve the treatment results for cancer patients. However, breast cancer patients still present a 20% to 50% prevalence of relapse over 10 years [2]. This is perhaps due to the natural consequences of radiation- or chemotherapy-related breast cancer treatment, which lead to micro- and macro-vasculature damage and then activation of several pro-angiogencic agents, thereby tumour cells can easily penetrate to the peripheral circulation and create a new tumour site in multi-step process [2,16]. Since relapse disease is caused by cancer dissemination, the terminal phase of cancer progression is accountable for most cancer-related deaths [2]. To date, little work has been undertaken to determine the impact of adjuvant treatment on the number of circulating endothelial progenitors in breast cancer patients. However, there are data that suggest that changes in circulating EPC levels may predict the efficacy of anticancer treatment. Unfortunately, the available data are sparse and controversial, probably due to the wide variety in the detection methods for circulating EPCs, non-standardised isolation techniques, and the various immune phenotypes of circulating EPCs, which were used to identify endothelial progenitors. It is well-established that the creation of a vascular network is a crucial step for tumour growth, invasion, and the spread to distant organs [1]. Circulating EPCs may be involved in tumour progression due to their ability to form new vessels and by secretion of paracrine agents that direct tumour cells to distant sites [7]. Additionally, circulating endothelial progenitors may reflect the level of vascular repair and neovascularisation [17].

It is widely expected that after surgery and cytotoxic therapy, the number of circulating EPCs will be reduced; this observation was confirmed by Kuo et al. [18] as well as the similar results that have been obtained in our study. We compared the number of circulating EPCs in patients after surgery and adjuvant treatment and found that both treatments reduced the number of circulating EPCs, but not to the level observed in healthy controls (Figure 1, Figure 2 and Figure 3). However, a relevant elevation in the number of circulating EPCs after nine months of treatment in the group of patients aged ≥ 55 years, of T2 clinical type, with nodal involvement (N1), and Ki67 expression ≥ 15% has been found in the current study. Increases in either the baseline or post-treatment circulating EPCs often, but not always, suggest an unfavourable response to anti-tumour therapy in cancer patients. 

Tanaka et al. believe that a lack of an increase in the number of circulating EPCs after the completion of the chemotherapy cycle is an independent negative prognostic factor. The authors suggest that changes in the number of circulating endothelial progenitor cells are associated with the response to treatment, since the mobilisation of EPCs may be associated with the adaptation of the body to chemotherapeutic agents. The authors claim that circulating endothelial progenitor cells can be a useful biomarker to monitor the adjuvant chemotherapeutic response [16]. Sakamori et al. suggest that selected chemotherapeutic patterns may induce mobilisation of EPCs from the bone marrow and a reduction in circulating EPC numbers is associated with the impaired endothelial function. Moreover, the authors have pointed out that EPC mobilisation after treatment is part of a host response mediated by the over-secretion of various cytokines involved in the recruitment of endothelial progenitors, such as stromal cell-derived factor 1 (SDF-1) [19]. Goon et al. demonstrated that certain types of chemotherapy and radiotherapy may be a major disruptive factor in clinical trials, as cytotoxic chemotherapy is specifically designed to attack the endothelium and damage it. Therefore, elevated von Willebrand factor plasma levels, with an increased number of circulating EPCs, may adequately reflect treatment response, but not necessarily the tumour spread [20]. Chemotherapy leads to direct endothelial dysfunction and it is associated with the essential mobilisation of the bone marrow-derived circulating EPCs in the circulation in order to substitute the impaired cells [16]. Stoelting et al. showed increased levels of circulating EPCs three weeks after the first cycle of dose-dense adjuvant chemotherapy. Chemotherapeutics, especially anthracyclines, can induce endothelial cell apoptosis resulting in an increase in the number of circulating EPCs and, consequently, circulating EPCs induce the vascular repair processes. The authors claim that it is unclear if the effect of this mobilisation of circulating EPCs is of biological relevance or is critical to the patient’s future outcome [21]. Based on Tanaka et al., Sakamori et al., and Goon et al.’s studies, we suggest that the lack of an increase in circulating EPCs after adjuvant treatment is a negative predictor for disease recurrence and worse future outcome [16,19,20]. In order to confirm this thesis, we demonstrate representative plots (Figure 2 and Figure 3), which present the individual flow cytometric analysis of two patients. One shows a case with a high pre-treatment level of circulating EPCs and an increase in the count after nine months of treatments. This circulating EPCs profile predicts a good prognosis after 40 months of observation. However, on the other hand, we show the circulating EPCs status in a premenopausal subject with a low baseline count of circulating EPCs with a further drop in the level of circulating EPCs. The 40-month follow-up revealed that after 27 months of follow-up, the patient developed a metastasis opposite axillary lymph nodes. Additionally, we can successfully compare and refer to the Tanaka et al. study due to the fact that these authors used the same immune phenotype as we did in order to identify circulating EPCs, hence the results might be easily interpreted and compared. 

It is worth noting that EPC-mediated neovascularisation depends on the type, stage, and size of the tumour. The behaviour of circulating EPCs is different in the early stage of breast cancer than in more advanced malignancies. The main purpose of chemotherapeutic agents is to induce cancer cell death. However, this type of therapy damages most of the cells, and also leads to endothelial dysfunction. However, certain cancer cells may survive and divide again, leading to the tumour spreading [7]. Interestingly, 40-month follow-ups revealed a significantly higher incidence of death and disease relapse in invasive breast cancer patients with low baseline circulating EPCs counts (<10.57 cells/μL) compared with those with higher pre-treatment numbers of circulating EPCs (≥10.57 cells/μL). The cut-off value of circulating EPCs count was established at 10.57 cells/μL, due to our previous findings, where we noted that the median value of circulating EPCs was 10.57 cells/μL in breast cancer patients as compared to healthy postmenopausal women as well as according to ROC analysis made in the current study, where the cut-off value was set at 10.55 cells/μL, which discriminates between the relapse disease group and the non-relapse disease patients [9]. This observation is compatible with our former investigations, since we observed that a lower number of circulating EPCs has been associated with higher tumour grading according to Elston–Ellis as well as with higher Ki67 expression [9,11]. We proposed in our previous paper that circulating endothelial progenitors are crucial agents at the early stage of tumour growth [9]. This theory was in line with Botelho et al.’s study. The authors imply that breast tumour cells mobilise EPCs in a precisely restricted way and demonstrate that the tumour involves EPCs during malignant transformation and that endothelial progenitors are no longer required when the tumour reaches a growth plateau [22]. Our study indicates that baseline circulating EPCs counts may serve as a non-invasive prognostic biomarker next to histological grading according to Elston–Ellis and Ki67 expression. Additionally, these results were supported by further statistical analyses (ROC curves, regression model), which also indicate that in patients with IBrC, next to tumour size and endocrine therapy, the number of circulating EPCs can predict the occurrence of disease relapse events or death. It is noteworthy that circulating EPCs, together with tumour diameter and Ki67 expression, distinguished IBrC patients with or without disease relapse (Figure 5). Pre-surgery circulating EPCs counts may help to identify patients at increased risk of future metastasis and can help to distinguish IBrC patients who responded well to treatment (chemotherapy/radiotherapy) and IBrC patients who did not respond to treatment. It is worth noting that the association between pre-treatment circulating EPCs number and disease relapse or death from neoplasm causes was independent of standard prognostic variables, such as tumour diameter, molecular subtype, and lymph node status, except for histological grading or the proliferative marker—Ki67. It has been demonstrated that lower counts of circulating EPCs may predict future cardiovascular events. In contrast, an increase in the EPC levels in the peripheral blood of patients with acute lung injuries led to an improved survival rate [7]. Shimoni et al. observed that low circulating EPCs levels were associated with cardiac death in patients with aortic stenosis (AS). They suggested that the lower numbers of circulating EPCs may be due to increased cell senescence as well as stimulation of circulating EPCs apoptosis in AS patients [23].

The present study has several limitations, including the relatively small number of patients. Thus, the results in this study could be biased and validation studies with a larger sample size are needed to confirm our results. In addition, the different molecular subtypes of IBrC were mixed in the same group; however, after excluding patients with luminal B HER2 negative and positive, non-luminal HER2 positive and basal-like subtype of IBrC circulating EPC levels were still comparable between patients with luminal A breast cancer. Consensus on the exact nature of EPCs is needed to create a standardised, generally accepted methodology and specific immune phenotype for enumeration of circulating EPCs.

## 5. Conclusions

Regardless of recent advances in the therapy of invasive breast cancer, there is still an unmet need of novel treatment strategies and the quest for non-invasive biomarkers for efficacy of adjuvant treatment and disease relapse. We suggest that quantification of circulating EPCs in peripheral blood may serve as a quick, inexpensive, and non-invasive biomarker for the monitoring of treatment response and tumour progression, in coexistence with other tumour-characteristic determinants already used. A further elucidation of the background underlying the behaviour of circulating EPCs in breast cancer patients and the function of EPCs in the renewal of vessels is of extreme importance for predicting cancerous patient survival.

## Figures and Tables

**Table 1:** The impact of adjuvant treatment on the number of circulating EPCs.

Feature	Baseline Blood Collection	Second Blood Collection	Third Blood Collection	*p*-Values
Circulating EPCs	10.15 4.84/23.72	3.62 2.12/5.73	6.17 2.10/10.37	**0.0004**
Age < 55 years	9.68 4.84/21.91	3.39 1.81/5.33	5.48 2.15/9.40	**0.0326**
Age ≥ 55 years	10.51 5.03/30.31	3.72 2.42/6.23	8.34 1.72/14.23	**0.0098**
Premenopausal	9.68 5.61/15.78	2.81 0.50/5.33	6.06 2.57/10.03	0.3679
Postmenopausal	10.50 4.82/30.23	3.79 2.51/6.13	6.44 1.95/11.19	**0.0006**
BMI < 24.9 kg/m^2^	7.84 4.34/26.07	4.12 2.16/6.49	6.01 1.78/13.31	0.2456
BMI ≥ 25 kg/m^2^	12.69 5.61/23.72	3.52 1.97/5.33	6.17 2.25/10.06	**0.0002**
Left breast cancer	11.04 5.03/23.72	3.87 2.51/6.13	5.22 1.82/10.06	**0.0098**
Right breast cancer	9.68 4.84/22.97	3.17 1.71/5.04	6.62 2.16/13.07	**0.0366**
Molecular subtypes Luminal A	10.55 5.23/23.72	3.76 2.52/6.18	6.17 1.89/12.17	**0.0116**
Other molecular subtypes	8.35 4.42/22.60	3.52 1.46/5.09	6.48 2.20/10.13	**0.0175**
TNM T1	10.70 4.94/25.18	3.52 2.51/5.33	5.72 1.82/9.79	**0.0011**
TNM T2	9.15 4.42/15.78	3.72 1.51/6.23	7.90 2.66/16.86	0.2335
Nodal status: N0	10.37 5.23/25.33	3.79 2.51/6.75	5.71 1.77/10.02	**0.0046**
N1	9.90 4.26/22.60	2.62 0.70/4.55	7.17 3.82/13.31	**0.0302**
Stage of disease IA	9.68 5.23/30.23	3.70 2.51/6.75	6.11 1.70/9.89	**0.0498**
Stage of disease IIA-IIB	10.15 4.42/15.78	3.72 1.51/5.04	6.04 2.33/13.54	**0.0085**
Expression of Ki67 < 15%	11.26 5.63/23.72	3.66 1.61/6.18	5.62 1.61/9.79	**0.0147**
Expression of Ki67 ≥ 15%	7.34 4.37/23.97	3.62 2.47/5.19	7.90 2.33/12.02	**0.0183**

EPCs: Endothelial progenitor cells; BMI: Body mass index; TNM—clinical classification: T—tumour, N—lymph node involvement, M—metastasis; Ki67: Proliferation marker; significant differences are denoted by bold *p*-values.

**Table 2:** Logistic regression analysis of progression-free predictors in breast cancer patients.

Variable	Code	Progression-Free Survival	
		OR (95% CI)	*p*-Value
Number of circulating EPCs	0–10.57 cells/μL >10.57 cells/μL	0.07 (0.01–0.67)	**0.0194**
Proliferative index Ki67	<15% ≥15%	1.43 (0.34–5.97)	0.6224
Tumour staging	IA IIA-IIB	4.23 (0.81–21.98)	0.0818
Tumour diameter	<2 cm ≥2 cm	10.24 (2.30–45.58)	**0.0019**
Oestrogen receptor status	Negative Positive	0.69 (0.04–11.29)	0.7982
Progesterone receptor status	Negative Positive	1.99 (0.17–22.88)	0.5766
Lymph node involvement	Positive Negative	1.97 (0.43–8.98)	0.3740
Chemotherapy	Yes No	0.39 (0.07–2.28)	0.2884
Brachytherapy	Yes No	0.68 (0.15–3.17)	0.6210
Surgery type	BCS MRM	2.33 (0.33–16.39)	0.3861
Endocrine therapy	Yes No	0.11 (0.01–0.93)	**0.0396**

OR: Odds ratio; CI: Confidence interval; EPCs: Endothelial progenitor cells; Ki67: Proliferation marker; significant differences are denoted by bold *p*-values; The underlined *p*-values represent closeness to statistical significance.
